# Machine-Learning-Assisted Instantaneous Frequency Measurement Method Based on Thin-Film Lithium Niobate on an Insulator Phase Modulator for Radar Detection

**DOI:** 10.3390/s24051489

**Published:** 2024-02-25

**Authors:** Qianqian Jia, Zichuan Xiang, Dechen Li, Jianguo Liu, Jinye Li

**Affiliations:** 1 Laboratory of Nano Optoelectronics, Institute of Semiconductors, Chinese Academy of Sciences, Beijing 100083, China; jiaqianqian@semi.ac.cn (Q.J.); xzc@semi.ac.cn (Z.X.); lidechen@semi.ac.cn (D.L.); jgliu@semi.ac.cn (J.L.); 2 College of Materials Science and Opto-Electronic Technology, University of Chinese Academy of Sciences, Beijing 100049, China

**Keywords:** microwave photonics, instantaneous frequency measurement, thin-film lithium niobate on insulator, stacking integrated learning

## Abstract

A simple microwave photonic, reconfigurable, instantaneous frequency measurement system based on low-voltage thin-film lithium niobate on an insulator phase modulator is put forward and experimentally demonstrated. Changing the wavelength of the optical carrier can realize the flexibility of the frequency measurement range and accuracy, showing that during the ranges of 0–10 GHz, 3–15 GHz, and 12–18 GHz, the average measurement errors are 26.9 MHz, 44.57 MHz, and 13.6 MHz, respectively, thanks to the stacked integrated learning models. Moreover, this system is still able to respond to microwave signals of as low as −30 dBm with the frequency measurement error of 62.06 MHz, as that low half-wave voltage for the phase modulator effectively improves the sensitivity of the system. The general-purpose, miniaturized, reconfigurable, instantaneous frequency measurement modules have unlimited potential in areas such as radar detection and early warning reception.

## 1. Introduction

The microwave photonics (MVP) technology-assisted instantaneous frequency measurement (IFM) system benefits from an ultrawideband operation, low loss, a fast response ability, and strong resistance to electromagnetic interference [1,2,3]. It has demonstrated extraordinary capabilities compared to traditional electronic measurement systems [4,5] in the areas of signal detection and early warning reception [6,7,8]. Over the past decade, new architectures for photonic-assisted instantaneous IFM systems have been proposed [9,10,11] and several common measurement theories have evolved, such as frequency–time mapping [12,13], frequency–space mapping [14,15,16], and frequency–power mapping [17]. In a different way, the core idea of these methods is to reflect unknown frequency information using physical quantities that a system can measure directly or indirectly.

A large measurement bandwidth (GHz) [18] and high measurement accuracy (MHz or even lower) [19] have been the goal of countless researchers worldwide. However, there is a mutual constraint relationship between measurement bandwidth and measurement accuracy. Different application scenarios put forward different requirements for the bandwidth, accuracy, sensitivity, and other indicators of the frequency measurement system. In recent years, various system architectures with different characteristics have been proposed for different application requirements [19,20,21,22,23,24,25].

To illustrate this, Di Wang [26] proposed an IFM system utilizing the principle of stimulated Brillouin scattering (SBS). Furthermore, the Brillouin gain–loss compensation is used to extend the measurement range. The experimental results show that the measurement range of this system can be extended from 20 GHz to 40 GHz with a higher accuracy of ~4 MHz, where *V*_B_ is the Brillouin frequency shift. Two years later, a photonic link [10] was raised, which can measure both frequency and phase information due to the introduce of a phase-varying reference signal based on SBS-induced carrier processing for an intercept receiver. The simulation results show that the system has a wide measurement range, from 0.2 to 18 GHz, and the measurement error is around 0.12 GHz. Moreover, the resolution of the received signal’s phase is 0.05 degrees over 0–2π. However, the complex system architecture constrains its practical application and lacks experimental data support. To realize the adjustable measuring bandwidth, Zahra Rabbani devised a simple IFM system with a dual output Sagnac loop [27], which has a 14 GHz measurement range and a less than 75 MHz error. However, the use of a Sagnac loop hinders the evolution of the system towards integration. To achieve a tunable measuring range and to improve system integration, a Mach–Zehnder interferometer (MZI) coupled ring array and a two-step measurement method [11] were introduced by Li Liu to establish a large bandwidth and high-precision frequency measurement. Thanks to the ultra-high peak rejection of the microwave photonic filter (MPF) response and flexibly tunable range, the system exhibits a measurement bandwidth of 40 GHz and a measurement accuracy of 9 MHz. Although the system exhibits excellent measurement performance, it requires precise tuning of the micro-ring electrodes and is sensitive to ambient temperature. On the other hand, Guodong Wang [28] put forward an IFM system that mainly consists of a dual-polarization quadrature phase shift keying modulator (DP-QPSKM) and a balanced photodetector (BPD) to improve the system’s robustness. Firstly, the use of balanced photodetection cancels out the beat components generated by the unknown frequencies themselves to exclude frequency misjudgment. Secondly, the relative time difference between the reference signal and the unknown frequency can avoid the measuring bias caused by time synchronization. However, again, the use of complex modulators makes it difficult to control the operating state of the system.

In brief, radar systems are developing in the direction of miniaturization and integration. For instantaneous frequency measurement systems, more compact dimensions, a simpler system construction, and the more flexible system tunability of different application scenarios are the direction of development. In addition, in cases where the performance of the frequency measurement hardware system is difficult to improve, computer models such as machine learning [29,30,31] can be used to optimize the measurement results and improve the robustness of the system. In this paper, a simple reconfigurable IFM system based on a low-voltage, thin-film, lithium niobate on insulator (LNOI) phase modulator (PM) for radar detection is exhibited, which features four advantages:

- The measurement system consists of an LNOI phase modulator and a wavelength division multiplexer (WDM) without the need for bias point control, with a simple and reliable architecture and a high degree of integration.
- LNOI phase modulators with low half-wave voltages provide higher sensitivity to the system and, in the future, will enable ultra-high-modulation bandwidths, further extending the measurement range.
- The tunability of carrier wavelength and filtering range can flexibly change the measurement range and measurement accuracy of the system to adapt to a wider range of scenarios.
- The use of stacked integrated learning models can further improve the system measurement’s accuracy and stability.

## 2. Theoretical Analysis

### 2.1. MWP IFM System Architecture and Principle

**Figure 1:**
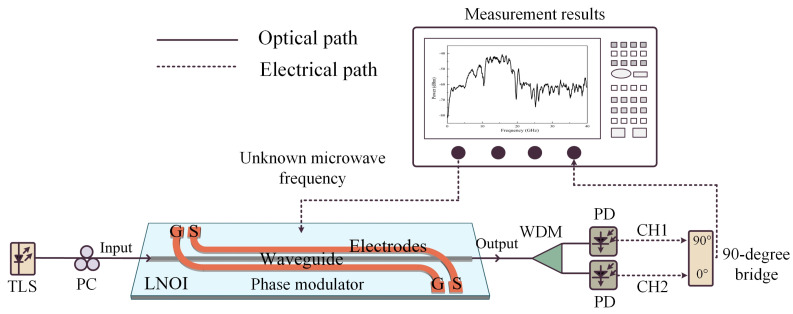
Architecture of a simple, reconfigurable IFM system based on an LNOI phase modulator. TLS: tunable laser source; PC: polarization controller; WDM: wavelength division multiplexer; PD: photodiode.

A schematic diagram of the photon-assisted IFM system architecture based on a thin-film lithium niobate phase modulator is shown in Figure 1. The light from the tunable laser source (TLS) source is injected into a lithium niobate phase modulator after a polarization controller (PC) to provide an optical carrier for the system, and its output light field can be written as follows:

(1) $$E_{0}\left(t\right)=E_{0}\exp\left(j\omega_{c}t\right)$$

where *E*_0_ is amplitude and *ω*_c_ is the angular frequency of TLS. A radio frequency (RF) signal of unknown frequency with amplitude *V*_RF_ and angular frequency *ω*_RF_ = 2π*f*_RF_ is loaded onto the phase modulator and the electrical signal can be described as follows:

(2) $$V_{RF}\left(t\right)=V_{RF}\cos\left(\omega_{RF}t\right)$$

The output optical field of the PM is derived as **follows**:

(3) $$E_{PM}\left(t\right)=E_{0}\exp\left[j\omega_{c}t+jm_{RF}\cos\left(\omega_{RF}t\right)\right]$$

where *m*_RF_ = π*V*_RF_/*V*_π_ is the RF signal’s modulation index and *V*_π_ is the PMs’ half-wave voltage. Expanding this using the Bessel function, the output light field of the PM can be written as follows:

(4) $$E_{PM}\left(t\right)=E_{0}\sum_{n=-\infty }^{\infty }J_{n}\left(m_{RF}\right)\exp\left(j\omega_{c}t+j\omega_{RF}t+jn\frac{\pi }{2}\right)$$

where *J*_±n_ () is the ±nst first kind of Bessel function. If only positive and negative first-order sidebands are considered, the above equation can be simplified as follows:

(5) $$E_{PM}\left(t\right)=E_{0}\left\{\begin{array}{l} J_{0}\left(m_{RF}\right)\cos\omega_{c}t+J_{1}\left(m_{RF}\right)\cos\left[\left(\omega_{c}+\omega_{RF}\right)t+\frac{\pi }{2}\right]\\ -J_{1}\left(m_{RF}\right)\cos\left[\left(\omega_{c}-\omega_{RF}\right)t-\frac{\pi }{2}\right] \end{array}\right\}$$

The modulated optical signal is split into two optical signals after passing through the dual-channel dense WDM. WDM is equivalent to a broadband dual-channel coarse filter, and the output optical signal of each channel contains a part of the carrier signal and positive first-order or negative first-order sidebands; the upper and lower output optical signals can be written as follows. Theoretically, the function of dual-channel WDM is to separate the positive and negative first-order sidebands of the modulated signal. However, due to the overlap between WDM channels, the output optical signal of each channel also includes a weaker optical carrier signal.

(6) $$\begin{array}{ll} E_{WDM}\left(t\right) & =\left[\begin{array}{l} E_{CH1}\left(t\right)\\ E_{CH2}\left(t\right) \end{array}\right]\\ & =\left\{\begin{array}{l} H_{CH1}\left(\omega_{c}\right)\left[J_{0}\left(m_{RF}\right)\exp\left(j\omega_{c}t\right)+J_{1}\left(m_{RF}\right)\exp\left(j\omega_{c}t+j\omega_{RF}t+\frac{\pi }{2}\right)\right]\\ H_{CH2}\left(\omega_{c}\right)\left[J_{0}\left(m_{RF}\right)\exp\left(j\omega_{c}t\right)-J_{1}\left(m_{RF}\right)\exp\left(j\omega_{c}t-j\omega_{RF}t-\frac{\pi }{2}\right)\right] \end{array}\right\} \end{array}$$

where H_CH1/2_() is the filter function introduced by WDM and is related to the center wavelength and filter bandwidth of WDM. The output optical signals of the two channels are connected to the broadband detectors, respectively. After PD detection, using *I*_out_∝*E*·E^*^, ignoring the high-order frequency components and DC components, the following can be established:

(7) $$\left[\begin{array}{l} I_{CH1}\left(t\right)\\ I_{CH2}\left(t\right) \end{array}\right]\propto \left[\begin{array}{l} E_{CH1}\left(t\right)E_{CH1}^{*}\left(t\right)\\ E_{CH2}\left(t\right)E_{CH2}^{*}\left(t\right) \end{array}\right]\propto \left[\begin{array}{l} J_{0}\left(m_{RF}\right)H_{CH1}^{2}\left(\omega_{c}\right)J_{1}\left(m_{RF}\right)\cos\left(\omega_{RF}t-\frac{\pi }{2}\right)\\ J_{0}\left(m_{RF}\right)H_{CH2}^{2}\left(\omega_{c}\right)J_{1}\left(m_{RF}\right)\cos\left(\omega_{RF}t+\frac{\pi }{2}\right) \end{array}\right]$$

The microwave powers after two PDs are proportional to the following:

(8) $$\left[\begin{array}{l} P_{CH1}\left(t\right)\\ P_{CH2}\left(t\right) \end{array}\right]\propto \left[\begin{array}{l} J_{0}^{2}\left(m_{RF}\right)H_{CH1}^{4}\left(\omega_{c}\right)J_{1}^{2}\left(m_{RF}\right)\sin^{2}\left(\omega_{RF}t\right)\\ J_{0}^{2}\left(m_{RF}\right)H_{CH2}^{4}\left(\omega_{c}\right)J_{1}^{2}\left(m_{RF}\right)\sin^{2}\left(\omega_{RF}t\right) \end{array}\right]$$

The two electrical signals pass through a 90-degree electrical coupler and the output current and electric field are written separately, as follows. This shows that the output electrical signal power is a function of the input RF signal frequency; thus, the unknown frequency information reacts to the power information that can be measured.

(9) $$\begin{array}{l} I_{out}\left(t\right)=\left[I_{CH1}\left(t\right)+\frac{\pi }{2}\right]+I_{CH2}\left(t\right)\\ \;\;\;\;\;\;\;\;\;\;\propto J_{0}\left(m_{RF}\right)J_{1}\left(m_{RF}\right)\left[H_{CH1}^{2}\left(\omega_{c}\right)\cos\left(\omega_{RF}t\right)-H_{CH2}^{2}\left(\omega_{c}\right)\sin\left(\omega_{RF}t\right)\right] \end{array}$$

(10) $$\begin{array}{l} P_{out}\left(t\right)\propto I^{2}{\;}_{out}\left(t\right)\\ \propto J_{0}^{2}\left(m_{RF}\right)J_{1}^{2}\left(m_{RF}\right)\left[\begin{array}{l} H_{CH1}^{4}\left(\omega_{c}\right)\cos^{2}\left(\omega_{RF}t\right)+H_{CH2}^{4}\left(\omega_{c}\right)-H_{CH2}^{4}\left(\omega_{c}\right)\cos^{2}\left(\omega_{RF}t\right)\\ -H_{CH1}^{2}\left(\omega_{c}\right)H_{CH2}^{2}\left(\omega_{c}\right)\sin\left(2\omega_{RF}t\right) \end{array}\right]\\ \propto J_{0}^{2}\left(m_{RF}\right)J_{1}^{2}\left(m_{RF}\right)\left\{\begin{array}{l} \frac{1}{2}\left[H_{CH1}^{4}\left(\omega_{c}\right)-H_{CH2}^{4}\left(\omega_{c}\right)\right]\cos\left(2\omega_{RF}t\right)\\ -H_{CH1}^{2}\left(\omega_{c}\right)H_{CH2}^{2}\left(\omega_{c}\right)\sin\left(2\omega_{RF}t\right) \end{array}\right\} \end{array}$$

**Figure 2:**
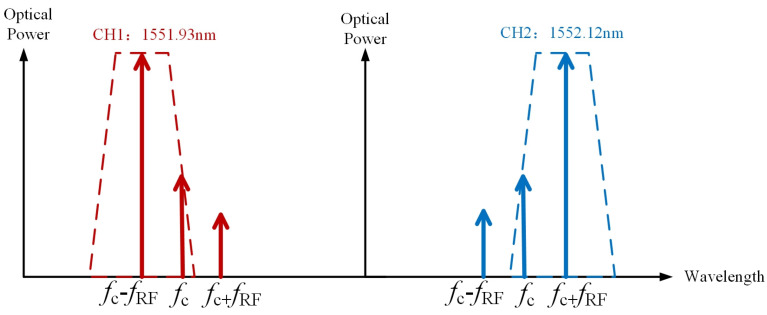
Schematic diagram of DWDM filtering effect with an RF signal close to the optical carrier.

As shown in Figure 2, dual-channel DWDM is used in the frequency measurement system. The center wavelengths of the sub-channels are 1551.93 nm and 1552.12 nm, respectively. The channel bandwidth is 50 GHz and the 1 dB bandwidth is 31 GHz. Due to the overlap between the two channels of DWDM, when the wavelength of the optical carrier is 1552 nm, the modulated optical signal with a positive and negative first-order sideband output achieved by the phase modulator passes through DWDM, the positive and negative first-order sideband output from the two channels, and the output optical signal of each subchannel also contains the optical carrier and the opposite side band with weak power at the RF signal close to the optical carrier. The intensity of the clutter signals is related to the filtering bandwidth and the steepness of the filtering edge. The DWDM is flat over a wide bandwidth range, so if the frequency measurement system used to achieve more accurate frequency measurements needs to replace other types of filters, the cascade micro-ring structure can be considered as constituting a tunable bandpass filter. This means that the application needs to be more flexible to adjust the filter bandwidth and the center wavelength. Combined with the frequency measurement feedback circuit, the center wavelength and bandwidth of the cascaded micro-ring filter can be adjusted to achieve a fast response measurement of RF signals at different frequencies. When the parameters of DWDM in the frequency measurement system are fixed, for RF signals of unknown frequency, the optical carrier wavelength is first adjusted to near the center wavelength of the two sub-channels of DWDM. At this time, the spectrum slope of the coupled output electrical signal is large, and can carry out rough measurements; then, the optical carrier wavelength is adjusted to the center of the DWDM to achieve more accurate frequency measurements.

**Figure 3:**
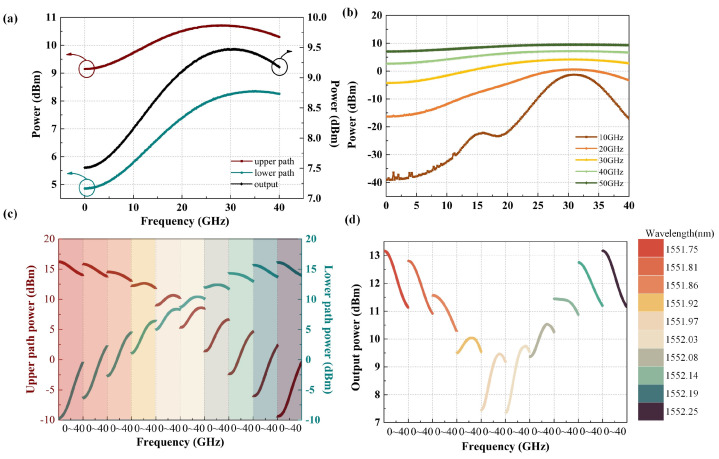
A series of simulation results of the proposed measurement system: (**a**) the spectral response of the upper and lower branches, as well as the 90-degree coupling output when the optical carrier wavelength is 1552 nm; (**b**) the frequency response of the 90-degree coupling output when the subchannel filtering bandwidths of the WDM are 10 GHz, 20 GHz, 30 GHz, 40 GHz, and 50 GHz, respectively; and (**c**,**d**) the spectral response of two branches, as well as the 90-degree coupling output at different optical carrier wavelengths.

The microwave photonic link shown in Figure 1 is built using Optisystem 15 optical path simulation software for simulation and analysis, and the emulation results of the frequency measurement are shown in Figure 3. The output optical carrier wavelength of TLS is 1552 nm, with an output power of 10 dBm, and the center wavelengths of the two channels of the DWDM are 1551.93 nm and 1552.12 nm, respectively, with a subchannel bandwidth of 50 GHz. Firstly, the frequency of the RF signal varies from 0 GHz to 40 GHz over a wide bandwidth, and the spectrum of both channels and coupled output after the 90-degree bridge are recorded at each frequency point shown in Figure 3a, showing that the power of the coupled output at different frequencies varies; hence, the frequency information is reflected in the power of the output electrical signal and can be to realize unknown frequency measurements. The spectrum response corresponds to the input signal frequency points one-to-one, thereby achieving the estimation of unknown frequency information. More importantly, Equation (10) shows that the frequency measurement range and accuracy can be flexibly changed by adjusting the filter function *H*_CH1/2_(). Specifically, as shown in Figure 3b, the optical carrier with the modulated RF signal passes through DWDM to separate the positive and negative first-order sidebands, and the two optical signals are restored to RF signals with different power levels after beating frequency, thus reflecting the frequency information of unknown RF signals and the power fluctuations in the electrical signal. The function of DWDM is to separate the positive and negative first-order sideband to obtain the target beat signal. When the input frequency of the radio frequency signal is low, the optical carrier and the positive and negative first-order sideband are difficult to separate through DWDM, and the beat signal output by the detector has a low power change. When the input RF signal frequency is higher or the filter bandwidth is smaller, the positive and negative first-order side bands are separated, and the beat signal power output of the two detectors is significantly changed, and the slope is larger; as a result, the frequency measurement is more accurate for the frequency measurement system.

When DWDM is fixed, the optical carrier wavelength can be adjusted to change the subchannel filtering effect, as shown in Figure 3c. When the input RF signal is scanned from 0 to 40 GHz and the optical carrier wavelength changes from 1551.75 nm to 1552.25 nm, the powers of the two paths’ beat signals, sent to the detectors by the upper and lower branches, are different. After the 90-degree electrical coupler processing, the power fluctuations in the coupled electrical signal fluctuate to adjust the frequency measurement accuracy in Figure 3d.

### 2.2. Design of Low-Voltage LNOI Phase Modulator

**Figure 4:**
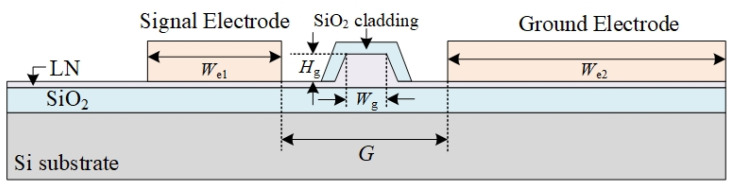
Design and parameterization of the phase modulator’s cross-section structure.

Thin-film lithium niobate combines the excellent electro-optical properties of lithium niobate with microfabrication processes to produce electro-optical modulators (EOM) with a compact size, low half-wave voltage, and high modulation bandwidth. In this article, the principles of EOM are based on the effect of the electric-field-induced refractive index change, which is called the Pockels effect [32,33]. When a signal voltage is applied to the waveguide, the effective refractive index of the waveguide varies with the electric field. To effectively utilize the maximum linear electro-optical coefficient (γ_33_ = 30.08 pm/V) of LN, the waveguide is x-cut y-transferred. The change in extraordinary refractive index due to the applied electric field can be written as follows:

(11) $$\Delta n_{e}=-\frac{1}{2}n_{e}^{3}\gamma_{33}E_{z}$$

where *E*_z_ denotes the electric field in the z-axis direction. In the *x*-cut LN thin film, half-wave voltage (*V*_π_) can be expressed as follows:

(12) $$V_{\pi }=\frac{n_{eff}\lambda G}{n_{e}^{4}\gamma_{33}\Gamma L}$$

where *n*_eff_ is effective refractive index; *G*, *L* and *Γ* are the electrode gap, the electrode length, and the overlap integral of the electric and optical fields, respectively. It is obvious that increasing the electrode length, decreasing the electrode spacing gap, and expanding the overlap integral of the electric and optical fields can reduce the half-wave voltage of the device. The design and dimensioning of the phase modulator cross-section is illustrated in Figure 4. The lithium niobate thin film is grown based on SOI sheets, and the thicknesses of the silicon substrate and silica buried oxygen are 300 µm and 2 µm, respectively. The thickness of the LN is 600 nm, the optical transmission is a ridge waveguide structure, and the width and height of the ridge waveguide are *W*_g_ and *H*_g_, respectively. An 800 nm thick silica cladding is covered on top of the ridge waveguide to limit the optical transmission and protect the waveguide structure, as well as to reduce the metal absorption loss. The phase modulator electrode adopts a traveling wave electrode structure and the 900 nm Au electrode is directly deposited on the LN flat waveguide; the widths of the signal electrode and the ground electrode are *W*_e1_ and *W*_e2_, respectively, and the lengths of the electrodes are both *L*. The spacing between the signal electrode and the ground electrode gap is *G*.

**Figure 5:**
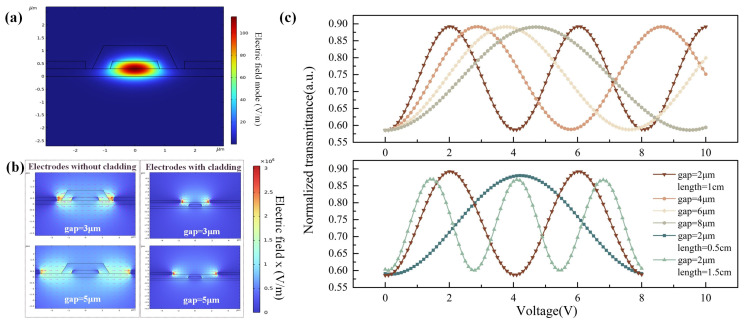
COMSOL-based simulation of the electric and optical field distributions of the phase modulator, (**a**) the electric field distribution in the waveguide cross-section of the phase modulator, (**b**) the electric field distribution with different electrode widths and cladding settings, and (**c**) the response of the modulator to the applied voltage with different electrode widths and electrode lengths.

The effect of the interaction between electric and optical fields in the waveguide was analyzed using COMSOL, as well as how to optimize the structural parameters of the phase modulator to achieve the reduction in the half-wave voltage, as shown in Figure 5. Based on the above structural design, the waveguide single-mode transmission mode field distribution is simulated as shown in Figure 5a, and the width and height of the ridge waveguide are optimized to determine the optimal single-mode transmission conditions of the waveguide. At the same time, in combination with the lithium niobate etching process, the height of the ridge waveguide *H*_g_ is set as 300 nm and the width of the ridge waveguide *W*_g_ is optimized to be 1.5 µm. To explore the effect of the interaction between the light field and the electric field, the electric field distributions of the waveguide and the electrode cross-section in various states are simulated in Figure 5b. It can be clearly seen that, in the case of the same electrode spacing, *G*, the ridge waveguide has a stronger electric field at the case of direct contact between the electrode and the LN (no cladding structure under the electrode); in addition, the electric field at the ridge waveguide is stronger when the electrode spacing *G* is closer. Combined with Equation (12), under the same conditions, the larger the electric field overlap integral, the lower the half-wave voltage. Therefore, the structure that retains the silica cladding above the ridge waveguide while the electrodes are in direct contact with the LN flat plate waveguide can be used to realize a low half-wave voltage. Moreover, the traveling wave electrode’s length also affects the modulator half-wave voltage, as shown in Figure 5c: with different combinations of electrode spacing *G* and electrode length *L*, the transmittance distributions of the phase modulator have different effects with the applied voltage. Theoretically, the transmittance distribution of the phase modulator varies sinusoidally, and the voltage difference between the adjacent maximum and minimum values is defined as the half-wave voltage. Simulation results show that the smaller the electrode spacing and the larger the electrode length, the smaller the modulator’s half-wave voltage. As the electrode spacing is too small and the process is difficult to realize, the longer the electrodes are, the more the modulator bandwidth decreases, and the finalized parameters are *L* = 1 cm and *G* = 6 μm when considering the fabrication tolerance.

On the other hand, as can be seen in Equation (12), the formula used to define the modulator half-wave voltage does not contain physical quantities related to the frequency information of the RF signal. Although the parameters of the material itself do not change with increasing frequency, there is inevitably a high-frequency attenuation in the RF portion of the waveguide, such as the electrodes, so that the RF half-wave voltage of the modulator increases with increasing frequency [34]. The use of modulators with low half-wave voltages ensures that the frequency measurement system has a modulated signal response with a higher signal-to-noise ratio to the input RF signal at high frequencies, thus improving the system’s sensitivity [35].

## 3. Results

### 3.1. Preparation and Characterization of LNOI Phase Modulator

**Figure 6:**
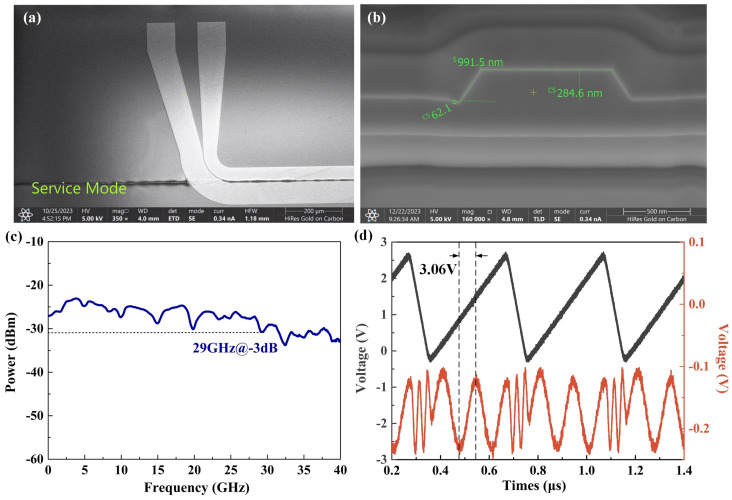
Performance characterization of the LNOI phase modulator. (**a**) and (**b**) represent SEM images of the electrodes and waveguide cross-section of the modulator; (**c**) electro-optical response S_21_ curve versus input frequency; (**d**) when the input signal is a 2 MHz sawtooth wave (black line), the modulator outputs an electrical signal waveform (red line).

Figure 6a,b show the cross-section and electrode constructions of the LNOI phase modulator observed by scanning electron microscopy (SEM). The phase modulator adopts electron beam lithography (EBL) combined with the lithium niobate dry-etching process to form a ridge waveguide structure, whose width and height are 1 μm and 300 nm, respectively, and the inclination of the waveguide cross-section is about 63 degrees. Furthermore, the top of the waveguide is covered with a SiO_2_ cladding of 800 nm. The prepared phase modulator is measured and tested for the main parametric indicators, which are half-wave voltage measurements and electro-optical modulation bandwidth, as shown in Figure 6c,d. It is worth noting that the phase modulator requires the construction of an interferometric link to test the electro-optical parameters. Figure 6c shows that the 3 dB bandwidth of the prepared phase modulator is 29 GHz and there is no steep drop in the signal in the range of 0–40 GHz, meaning that the operation band is about 35 GHz. The half-wave voltage of the phase modulator is 3.06 V@2 MHz in Figure 6d. In Figure 6d, the triangular wave signal is still continuously varying in voltage at the inflection point, and by applying such a signal at the RF electrodes of the phase modulator, the response of the phase modulator is still approximately sinusoidal, with a maximum and a minimum. This also indicates that the phase modulator response is faster compared to the injected triangular wave signal period.

### 3.2. Instantaneous Frequency Measurement and Optimization

The experimental system is built according to the schematic diagram shown in Figure 1. The output power of TLS (TSL-570) is 10 dBm, with wavelengths varying from 1552.12 nm to 1552.92 nm; the vector network analyzer (VNA, N5227B) is used to provide a microwave RF signal and inject it into PM to produce a modulated optical signal; the modulated optical signal is beam-split into two optical signals after dual-channel DWDM and injected into the parallel PDs (XPDV2150R, XPDV3120R), which produce two microwave signals. Subsequently, after a 90-degree bridge coupler, the final output signal is obtained. VNA is set to sweep from 0.1 GHz to 40 GHz with the power 0 dBm and receive microwave signal responses.

**Figure 7:**
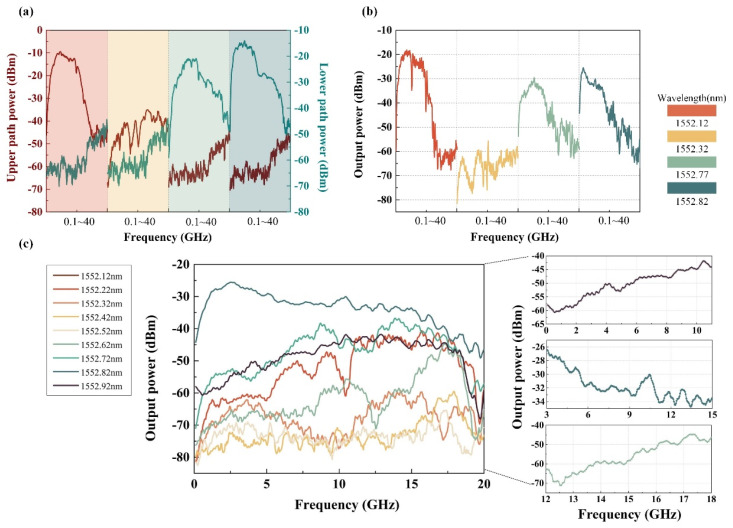
Present experimental results of frequency measurements of the proposed system. (**a**) and (**b**) represent the spectral responses of the upper and lower branches, as well as the 90-degree coupled outputs, when the optical carrier wavelengths are 1552.12 nm, 1552.32 nm, 1552.77 nm, and 1552.82 nm, respectively; (**c**) spectral responses of the 90-degree coupled outputs for different optical carrier wavelengths with the three frequency-measured monotonic intervals.

**Figure 8:**
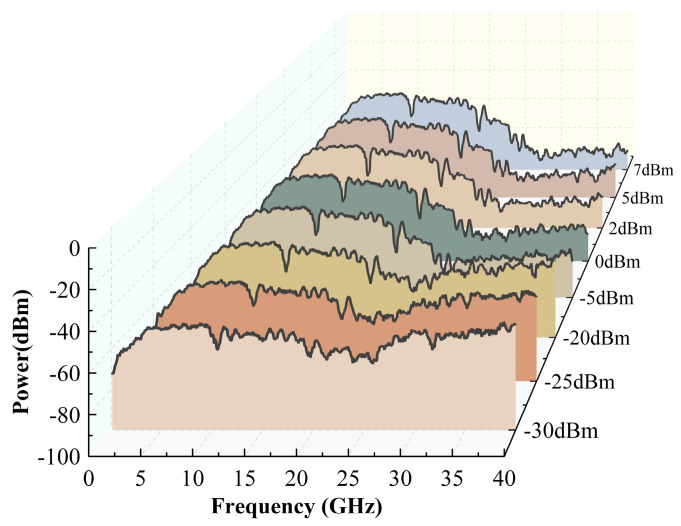
Spectral response of 90-degree coupled output at different input microwave signal RF powers when the optical carrier wave wavelength is 1552.12 nm.

The microwave photonic link is built based on the frequency measurement system architecture shown in Figure 1, and the experimental results are shown in Figure 7. Firstly, when the TLS output wavelengths are 1552.12 nm, 1552.32 nm, 1552.77 nm, and 1552.82 nm, the RF signal takes 400 frequency points equidistantly from the range of 0.1 GHz–40 GHz and injects them into the frequency measurement system. The spectrum response of the beat frequency electrical signal of the upper and lower branches is shown in Figure 7a. When the optical carrier wavelength is located near the center of the wavelength of the two DWDM channels, the difference in the fluctuations in the electrical signals of the two branches is not obvious; when the optical carrier is biased toward the center of the wavelength of a certain channel, the fluctuations in the electrical signal of the channel change significantly, since DWDM coarse filtering destroys the sideband symmetry of the phase modulator. Furthermore, due to the different fluctuations in the electrical signals of the two circuits under different optical carrier states, the spectrum response of the final electrical signal output through the 90-degree electrical coupler also has similar changes, as shown in Figure 7b. Different spectrum response slopes are suitable for frequency measurements with different levels of accuracy. In addition, upon setting the wavelength of TLS to range from 1552.12 nm to1552.92 nm, the electrical spectrums of the different coupling outputs are displayed in Figure 7c. Although there is jitter in the measured spectrum response, the trend of the results is consistent with the simulation results (Figure 3c,d). When the optical carrier wavelength is located at the center of the wavelength of the two DWDM channels, the final coupled output electrical spectrum response fluctuations are relatively gentle; when the optical carrier wavelength moves between the two DWDM channels, the final output electrical spectrum slope changes greatly. When the optical carriers are 1552.12 nm, 1552.82 nm, and 1552.62 nm, respectively, three monotonic intervals with large slopes of 0–10 GHz, 3–15 GHz, and 12–18 GHz are selected for further frequency measurement effect analysis in Figure 7c. Figure 8 shows that the IFM system is still able to respond normally when the power of the input microwave frequency signal is as low as −30 dBm, which demonstrates better sensitivity thanks to the use of a low half-wave voltage phase modulator.

**Figure 9:**
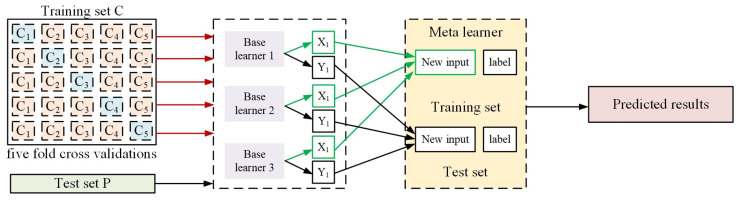
Stacking ensemble learning model.

**Figure 10:**
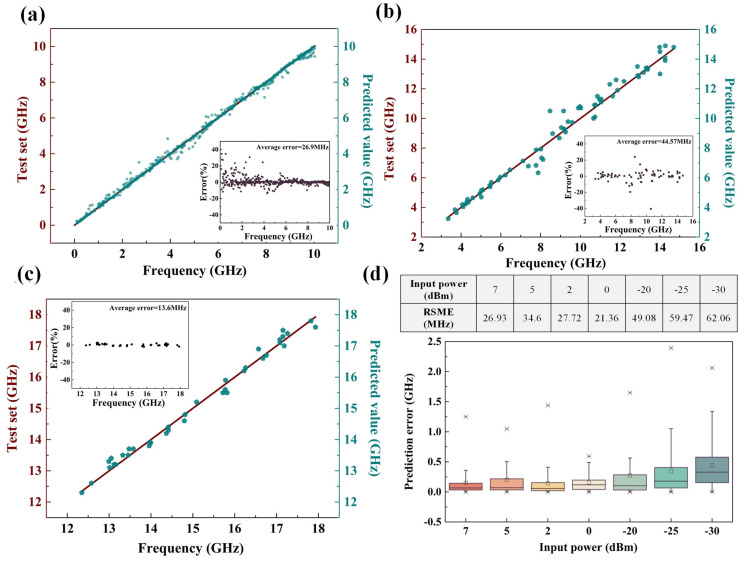
Optimization results and average measurement errors of frequency measurement under different monotonic intervals: (**a**) 0–10 GHz, (**b**) 3–15 GHz, and (**c**) 12–18 GHz. (**d**) Analysis of frequency measurement error results under virous input microwave signal powers when the monotonic interval is 0–10 GHz.

The stacking integrated learning method is a method that combines theoretical knowledge of statistics with machine learning algorithms [31,36,37], whose framework principle is shown in Figure 9. The stacked integrated learning method combines multiple single machine learning algorithms (base learners) and high-level learners (meta-learners) to leverage the advantages of each algorithm to obtain a better prediction performance, which can further improve the measurement accuracy on the condition that the hardware system cannot be further optimized. The optimized frequency measurement results after applying this method to the proposed frequency measurement system are shown in Figure 10a–d. When the optical carrier wavelength is 1552.12 nm; the frequency measurement error in the monotonic interval 0–10 GHz is calculated. The input test signal is shown in Table A1. The output prediction value, optimized with the help of the stacked integrated learning model, is obtained, and the measurement error is calculated with the value of 26.9 MHz, as shown in Figure 10a. Similarly, when the optical carrier wavelength is 1552.82 nm, the frequency measurements in the monotonic interval 3–15 GHz are evaluated, the input test signal and the predicted output signal frequencies are shown in Table A2, and, at this time, the average measurement error is 44.57 MHz, as shown in Figure 10b. When the optical carrier wavelength is 1552.62 nm, the average error of the frequency measurement in the monotonic interval of 12–18 GHz is 13.6 MHz in Figure 10c, and the specific measurement data are shown in Table A3. In addition, when the RF signal input power is 7 dBm, 5 dBm, 2 dBm, 0 dBm, −20 dBm, −25 dBm, and −30 dBm, respectively, the frequency measurement results of the same monotonic interval 0–10 GHz are evaluated, as shown in Figure 10d, and the frequency measurement error increases as the input RF signal power decreases. However, in general, when the input RF signal power is as low as −30 dBm, the measurement system still responds normally, and the root mean square error (RSME) is 62.06 MHz.

## 4. Discussion

In summary, this paper proposes a simple reconfigurable IFM system based on a low-voltage LNOI phase modulator for radar detection, which demonstrates high sensitivity, integrated miniaturization, and an adjustable measurement range and high measurement accuracy with the help of stacked integrated learning models compared with the high-performance discrete microwave photonic systems and the on-chip structures that have been reported. For instance, the IFM systems proposed in [11,27] are both capable of achieving reconfigurable measurement ranges and accuracies. However, the structure in [27] is based on a Sagnac loop, making it difficult to achieve on-chip integration, while the on-chip MZ interferometric structure [11] relies on the precise control of the thermal electrodes and is sensitive to the ambient temperature. The proposed work uses LNOI material, as well as exhibiting a suitable sensitivity and tunable measurement range accuracy, with no need to control the work point to adapt it to the needs of various application scenarios (Table 1).

## 5. Conclusions

As a result, a simple, reconfigurable IFM system based on the use of a low-voltage LNOI phase modulator for radar detection is proposed for radar detection, with high reliability without bias point control, integration, an excellent sensitivity with the help of half-wave voltage, small measurement error when using stacked integrated learning models, and tunable measurement parameters for a wider range of scenarios. The advantage of this system is that this is the first time that the LNOI phase modulator is used in a frequency measurement system, proving to be simple and reliable, and the measurement results are optimized by combining the stacked integrated learning model to meet the different needs of rough measurements and precise measurements. In the future, the system is expected to be integrated, as DWDM can be replaced by a cascaded micro-ring structure to achieve on-chip multi-functional integration. The sufficient experimental results show that the system can realize frequency measurements in adjustable ranges, such as 0–10 GHz, 3–15 GHz, and 12–18 GHz, with average measurement errors of 26.9 MHz, 44.57 MHz, and 13.6 MHz, respectively. The sensitivity of this system is superior, showing that when the measured signal power is as low as −30 dBm, the average measurement error is 62.06 MHz. An integrated, reconfigurable, and robust instantaneous frequency measurement system based on the LNOI phase modulator will meet the needs of future array radar detection.

## Figures and Tables

**Table 1:** Performance comparison between the proposed system and reported methods.

Device/Structure	Range	Error	Sensitivity	Tunability
PM+DPMZM+SMF+MZM [26]	20–40 GHz	4 MHz	-	×
DPMZM+DM [21]	5–15 GHz	12 MHz	-	×
DP-QPSKM [28]	16–26 GHz	7.53 MHz	-	×
PM+ Sagnac loop [27]	0–14 GHz	75 MHz	5 dBm	√
Si micro-disks [19]	1.6–40 GHz	60 MHz	-	×
Si MRR [23]	0.5–35 GHz	300 MHz	-	×
Si MZI [11]	0–40 GHz	9 MHz	-	√
	0–20 GHz	4 MHz		
Si3N4 MRR [38]	0.5–4 GHz	93.6 rms	−3 dBm	×
LNOI PM (this work)	0–10 GHz	26.9 MHz	−30 dBm	√
	3–15 GHz	44.57 MHz		
	12–18 GHz	13.6 MHz		

## Data Availability

The data presented in this study are available on request from the corresponding author.

## Appendix A

The frequency measurement microwave photonic structure proposed in this paper belongs to a point-by-point scanning frequency measurement system. The broadband signal generated by the vector network analyzer is injected into the system to obtain the coupled output broadband electrical spectrum response curve. The more frequency points the broadband signal uses, the closer the obtained spectrum response curve is to the real situation. Later, when a signal of unknown frequency is injected into the measurement system, more coupled output electrical signal power can be used to deduce the frequency point of the input signal, thereby achieving a wide range of unknown signal frequency point measurements. Its measurement speed is consistent with the response of the modulator, the detector, and other optoelectronic devices.

Before evaluating the frequency measurement effect of this frequency measurement system, a vector network analyzer is used to inject the system with RF signals of 1000 frequency points, taken at equal spacing in the range of 0–40 GHz, to obtain the spectra of the upper and lower branches, as well as the final coupled output electrical signals at different optical carrier wavelengths, which are used for model training and optimization. The appendix details the raw data of the frequency measurements (Table A1, Table A2 and Table A3) for the three monotonic intervals of 0–10 GHz, 3–15 GHz, and 12–18 GHz, including the input test frequencies, the output predictions, and the percentage of measurement errors.

**Table A1:** Frequency measurement results and error percentage in the range of 0–10 GHz.

Input Signal (Hz)	Output Signal (Hz)	Measurement Error Percentage	Input Signal (Hz)	Output Signal (Hz)	Measurement Error Percentage
10,025,100,000	10,023,736,586	0.01%	7,381,750,000	7,321,957,825	0.81%
5,586,250,000	5,579,099,600	0.13%	8,429,130,000	8,388,670,176	0.48%
5,386,750,000	5,382,117,395	0.09%	9,177,250,000	9,157,427,140	0.22%
8,628,630,000	8,628,529,045	0.00%	10,025,100,000	9,908,608,338	1.16%
698,500,000	684,781,460	1.96%	3,691,000,000	3,684,208,560	0.18%
2,643,630,000	2,617,273,009	1.00%	6,284,500,000	6,277,712,740	0.11%
6,583,750,000	6,583,522,202	0.00%	9,376,750,000	9,328,365,970	0.52%
748,375,000	747,938,697.4	0.06%	6,683,500,000	6,681,220,927	0.03%
2,693,500,000	2,677,500,610	0.59%	4,838,130,000	4,834,051,955	0.08%
9,875,500,000	9,882,847,372	0.07%	4,937,880,000	4,937,857,016	0.00%
9,975,250,000	9,974,608,591	0.01%	8,429,130,000	8,413,030,362	0.19%
7,531,380,000	7,463,974,149	0.90%	2,045,130,000	2,041,775,987	0.16%
2,444,130,000	2,437,481,966	0.27%	3,591,250,000	3,551,530,775	1.11%
5,536,380,000	5,521,963,266	0.26%	7,381,750,000	7,377,151,170	0.06%
3,890,500,000	3,866,106,565	0.63%	4,439,130,000	4,438,810,383	0.01%
3,591,250,000	3,581,661,363	0.27%	3,192,250,000	3,191,835,008	0.01%
299,500,000	297,921,635	0.53%	6,583,750,000	6,578,575,396	0.08%
7,331,880,000	7,331,672,508	0.00%	4,489,000,000	4,317,520,200	3.82%
2,494,000,000	2,502,030,680	0.32%	3,691,000,000	3,547,863,020	3.88%
9,127,380,000	9,219,110,169	1.01%	6,234,630,000	6,205,950,702	0.46%
8,229,630,000	8,379,656,155	1.82%	3,591,250,000	3,590,136,713	0.03%
2,543,880,000	2,564,688,938	0.82%	7,681,000,000	7,678,020,747	0.04%
7,730,880,000	7,738,343,152	0.10%	9,626,130,000	9,597,636,655	0.30%
3,192,250,000	3,171,723,833	0.64%	9,127,380,000	9,108,851,419	0.20%
2,344,380,000	2,257,989,597	3.69%	4,489,000,000	4,465,073,630	0.53%
5,885,500,000	5,833,060,195	0.89%	7,481,500,000	7,488,173,498	0.09%
7,331,880,000	7,331,617,519	0.00%	9,426,630,000	9,440,392,880	0.15%
9,875,500,000	9,871,508,619	0.04%	8,878,000,000	8,930,824,100	0.60%
3,142,380,000	3,133,644,184	0.28%	4,638,630,000	4,655,329,068	0.36%
2,593,750,000	2,593,573,625	0.01%	3,341,880,000	3,346,346,810	0.13%
8,479,000,000	8,543,779,560	0.76%	6,434,130,000	6,421,326,081	0.20%
4,987,750,000	5,001,266,803	0.27%	3,990,250,000	3,990,018,566	0.01%
3,940,380,000	3,971,193,772	0.78%	7,531,380,000	7,531,003,431	0.01%
5,985,250,000	5,988,676,502	0.06%	5,486,500,000	5,486,009,507	0.01%
9,326,880,000	9,352,435,651	0.27%	6,234,630,000	6,228,038,126	0.11%
7,681,000,000	7,604,420,430	1.00%	2,244,630,000	2,244,374,786	0.01%
8,828,130,000	8,776,838,565	0.58%	8,329,380,000	8,316,611,060	0.15%
6,533,880,000	6,493,173,928	0.62%	9,925,380,000	9,809,848,577	1.16%
499,000,000	494,284,450	0.95%	6,234,630,000	6,224,841,631	0.16%
8,179,750,000	8,162,572,525	0.21%	6,783,250,000	6,910,368,105	1.87%

**Table A2:** Frequency measurement results and error percentage in the range of 3–15 GHz.

Input Signal (Hz)	Output Signal (Hz)	Measurement Error Percentage	Input Signal (Hz)	Output Signal (Hz)	Measurement Error Percentage
9,324,750,000	9,366,804,622.5	0.45%	13,313,700,000	13,399,972,776	0.65%
8,474,870,000	8,550,804,835.2	0.90%	9,381,370,000	9,404,729,611.30	0.25%
13,386,100,000	13,472,306,484	0.64%	7,771,020,000	7,779,490,411.80	0.11%
9,593,860,000	9,634,058,273.4	0.42%	9,200,200,000	9,230,008,648.00	0.32%
13,178,400,000	13,256,811,480	0.60%	9,193,210,000	9,279,993,902.40	0.94%
5,019,690,000	5,039,618,169.30	0.40%	14,254,700,000	14,242,583,505	0.09%
4,309,780,000	4,346,887,205.80	0.86%	14,267,700,000	14,185,375,371	0.58%
10,957,900,000	10,971,268,638	0.12%	10,714,700,000	10,636,482,690	0.73%
8,049,770,000	8,125,518,335.7	0.94%	4,105,320,000	4,081,057,558.80	0.59%
9,046,170,000	9,108,588,573	0.69%	7,778,310,000	7,754,430,588.30	0.31%
9,978,090,000	10,001,738,073.3	0.24%	5,973,280,000	5,966,291,262.40	0.12%
7,877,340,000	7,927,991,296.2	0.64%	4,327,360,000	4,287,807,929.60	0.91%
8,605,630,000	8,635,749,705	0.35%	5,098,780,000	5,007,562,825.80	1.79%
11,870,600,000	11,900,039,088	0.25%	9,260,410,000	9,348,198,686.80	0.95%
12,895,100,000	12,899,987,758.7	0.04%	11,630,500,000	11,760,994,210	1.12%
8,016,730,000	8,031,320,448.60	0.18%	12,877,200,000	12,984,853,392	0.84%
11,484,500,000	11,496,099,345	0.10%	5,444,730,000	5,489,975,706.30	0.83%
5,463,150,000	5,481,670,078.50	0.34%	10,019,500,000	9,940,245,755.00	0.79%
13,989,700,000	14,100,498,424	0.79%	4,232,630,000	4,225,265,223.80	0.17%
9,259,720,000	9,291,295,645.2	0.34%	5,871,120,000	5,840,003,064.00	0.53%
8,123,120,000	8,131,202,504.4	0.10%	4,064,540,000	4,061,003,850.20	0.09%
7,115,480,000	7,100,964,420.8	0.20%	5,520,600,000	5,511,270,186.00	0.17%
12,197,400,000	12,138,730,506	0.48%	9,899,990,000	9,891,971,008.10	0.08%
13,375,600,000	13,351,256,408	0.18%	11,801,200,000	11,698,647,572	0.87%
4,591,680,000	4,543,375,526.4	1.05%	6,460,430,000	6,455,455,468.90	0.08%
14,012,900,000	14,009,705,058.8	0.02%	3,768,740,000	3,753,062,041.60	0.42%
14,007,000,000	13,934,163,600	0.52%	12,937,600,000	12,945,880,064	0.06%
3,356,200,000	3,340,694,356	0.46%	14,279,200,000	14,229,508,384	0.35%
6,225,540,000	6,179,969,047.2	0.73%	3,694,690,000	3,660,218,542.30	0.93%
10,560,400,000	10,616,686,932	0.53%	4,365,560,000	4,340,021,474.00	0.59%
10,664,800,000	10,639,951,016	0.23%	11,051,600,000	11,024,192,032	0.25%
10,776,200,000	10,760,143,462	0.15%	8,943,570,000	8,858,874,392.10	0.95%
10,737,600,000	10,636,881,312	0.94%	7,384,570,000	7,363,376,284.10	0.29%
5,019,300,000	4,991,141,727	0.56%	4,951,410,000	4,967,749,653.00	0.33%
11,041,200,000	10,982,350,404	0.53%	14,699,300,000	14,711,794,405	0.09%
9,158,090,000	9,098,287,672.3	0.65%	10,858,600,000	11,065,673,502	1.91%
9,324,750,000	9,366,804,622.5	0.45%	13,313,700,000	13,399,972,776	0.65%

**Table A3:** Frequency measurement results and error percentage in the range of 12–18 GHz.

Input Signal (Hz)	Output Signal (Hz)	Measurement Error Percentage	Input Signal (Hz)	Output Signal (Hz)	Measurement Error Percentage
15,774,400,000	15,776,072,086	0.01%	14,416,400,000	14,404,765,965	0.08%
17,823,900,000	17,826,288,403	0.01%	13,326,600,000	13,286,486,934	0.30%
13,972,600,000	13,979,865,752	0.05%	16,756,600,000	16,750,936,269	0.03%
14,414,700,000	14,429,402,994	0.10%	16,675,700,000	16,668,129,232	0.05%
17,184,200,000	17,185,437,262	0.01%	14,368,100,000	14,343,674,230	0.17%
13,052,900,000	13,053,760,186	0.01%	13,958,800,000	13,956,873,686	0.01%
17,153,000,000	17,167,700,121	0.09%	17,153,500,000	17,150,069,300	0.02%
17,079,200,000	17,077,992,501	0.01%	16,227,100,000	16,226,829,007	0.00%
13,013,600,000	13,004,958,970	0.07%	16,571,700,000	16,573,325,684	0.01%
13,580,400,000	13,568,435,668	0.09%	13,994,900,000	14,004,388,542	0.07%
12,576,900,000	12,553,758,504	0.18%	15,782,800,000	15,810,309,420	0.17%
15,820,200,000	15,823,996,848	0.02%	13,117,700,000	13,125,937,916	0.06%
17,263,700,000	17,277,321,059	0.08%	14,799,300,000	14,804,435,357	0.03%
12,995,600,000	13,040,044,952	0.34%	16,262,900,000	16,259,192,059	0.02%
13,477,400,000	13,389,527,352	0.65%	12,349,000,000	12,331,760,796	0.14%
15,091,800,000	15,080,979,179	0.07%	15,720,700,000	15,714,348,837	0.04%
14,814,300,000	14,800,016,837	0.10%	13,158,400,000	13,162,571,213	0.03%
17,931,300,000	17,916,112,189	0.08%	17,083,300,000	17,083,132,513	0.00%
13,454,300,000	13,408,555,380	0.34%			
